# Association between anthropization and rodent reservoirs of zoonotic pathogens in Northwestern Mexico

**DOI:** 10.1371/journal.pone.0298976

**Published:** 2024-02-22

**Authors:** Hugo Mendoza, Andrés M. López-Pérez, André V. Rubio, Julio J. Barrón-Rodríguez, Marisa Mazari-Hiriart, Paulina A. Pontifes, Rodolfo Dirzo, Gerardo Suzán

**Affiliations:** 1 Laboratorio de Ecología de Enfermedades y Una Salud, Departamento de Etología, Fauna Silvestre y Animales de Laboratorio, Facultad de Medicina Veterinaria y Zootecnia, Universidad Nacional Autónoma de México, Ciudad de México, México; 2 Laboratorio Nacional de Ciencias de la Sostenibilidad, Instituto de Ecología, Universidad Nacional Autónoma de México, Ciudad de México, México; 3 Department of Medicine and Epidemiology, School of Veterinary Medicine, University of California, Davis, CA, United States of America; 4 Red de Biología y Conservación de Vertebrados, Instituto de Ecología A.C., Xalapa, México; 5 Departamento de Ciencias Biológicas Animales, Facultad de Ciencias Veterinarias y Pecuarias, Universidad de Chile, Santiago, Chile; 6 MIVEGEC Unit, IRD, CNRS, Université de Montpellier, Montpellier, France; 7 Departments of Biology and Earth Systems Science, Stanford University, Stanford, CA, United States of America; National Veterinary Research Institute (NVRI), NIGERIA

## Abstract

The world is facing a major pulse of ecological and social changes that may favor the risk of zoonotic outbreaks. Such risk facilitation may occur through the modification of the host’s community diversity and structure, leading to an increase in pathogen reservoirs and the contact rate between these reservoirs and humans. Here, we examined whether anthropization alters the relative abundance and richness of zoonotic reservoir and non-reservoir rodents in three Socio-Ecological Systems. We hypothesized that anthropization increases the relative abundance and richness of rodent reservoirs while decreasing non-reservoir species. We first developed an Anthropization index based on 15 quantitative socio-ecological variables classified into five groups: 1) Vegetation type, 2) Urbanization degree, 3) Water quality, 4) Potential contaminant sources, and 5) Others. We then monitored rodent communities in three regions of Northwestern Mexico (Baja California, Chihuahua, and Sonora). A total of 683 rodents of 14 genera and 27 species were captured, nine of which have been identified as reservoirs of zoonotic pathogens (359 individuals, 53%). In all regions, we found that as anthropization increased, the relative abundance of reservoir rodents increased; in contrast, the relative abundance of non-reservoir rodents decreased. In Sonora, reservoir richness increased with increasing anthropization, while in Baja California and Chihuahua non-reservoir richness decreased as anthropization increased. We also found a significant positive relationship between the anthropization degree and the abundance of house mice (*Mus musculus*) and deer mice (*Peromyscus maniculatus*), the most abundant reservoir species in the study. These findings support the hypothesis that reservoir species of zoonotic pathogens increase their abundance in disturbed environments, which may increase the risk of pathogen exposure to humans, while anthropization creates an environmental filtering that promotes the local extinction of non-reservoir species.

## Introduction

The world is facing a critical stage of ecological and social changes that jeopardize the processes of Socio-Ecological Systems (SES) [1, 2]. These changes have been associated with several environmental and health issues, such as climate change and disease outbreaks [3–6]. One of the main drivers of this crisis is anthropization (i.e., changes in the environment due to human activities), including land-use change or environmental contamination [7–9]. Anthropization is a complex process in which diverse ecological, social, and economic variables interact with each other within a SESs [10, 11]. All these changes have increased contact rates between humans and pathogens associated with animal reservoirs by modifying species assemblages and behavior, increasing the risk of zoonotic burden [6, 8, 12–14].

Among all mammalian reservoirs of zoonotic pathogens, rodents (Rodentia) represent the most important Order with more than 80 zoonotic pathogens reported across different species [15–17]. Many studies have postulated that the capacity of rodents to serve as primary reservoirs of several pathogens is explained by their life-history traits (e.g., high litter size, number of litters per year) and their adaptability to anthropization [18–20]. In fact, land-use change and habitat fragmentation are the main processes that have been linked to increased rodent reservoir abundance [21, 22]. For example, *Rattus rattus*, *R*. *norvegicus*, and *Mus musculus* are species that benefit from human activities, and they are also reservoirs of pathogens of human health concern such as *Leptospira* spp., Seoul virus, and Lymphocytic Choriomeningitis virus [5, 15, 23, 24]. Thus, it is crucial to address the emergency related to anthropization and rodent reservoirs under a holistic approach, such as the One Health approach, which considers environmental, animal, and human health as interdependent [25]. One Health approach can be used not only to prevent future zoonoses but also to safeguard the natural integrity of ecosystems in a sustainable manner [26].

The complex association between rodent reservoirs and anthropization has been addressed in a few studies, mostly conducted in tropical regions, either by theoretical approaches (e.g., meta-analysis) [13, 21, 27] or through field studies that monitored pathogen prevalence in different animal species [28]. In the second case, there are certain limitations due to the use of qualitative variables, leading to bias due to the subjectivity of the researcher, and in the case of studies that used quantitative characteristics, they used a reduced number of variables, leaving information to chance [29–31]. In addition, there are other variables associated with anthropization that have not been investigated in relation to rodent reservoir abundance, which could be useful for understanding the effect of human activities on animal communities’ composition and the population dynamics of rodent reservoirs and non-reservoirs. For example, the presence of potential contaminant sources that may serve as a pathogen source, food resources for synanthropic reservoir species, or act as drivers of immunosuppressant, have not been associated with the dynamics of rodent reservoir species [19, 32].

In this study, we evaluated whether anthropization impacts the richness and abundance of reservoir and non-reservoir rodents in arid and semiarid SESs in Northwestern Mexico. We hypothesize that the richness and abundance of rodent reservoirs will increase as anthropization increases, while the richness and abundance of non-reservoir species will decrease. To test this hypothesis, we first monitored rodent abundance and richness and identified species previously reported as reservoirs of directly transmitted zoonotic pathogens; then, we developed an index based on socio-ecological characteristics to rate the anthropization degree of each study site within the region. Finally, we analyzed the statistical association between the richness and abundance of rodent reservoirs and non-reservoirs with the anthropization degree.

## Methods

### Study sites

**Fig 1:**
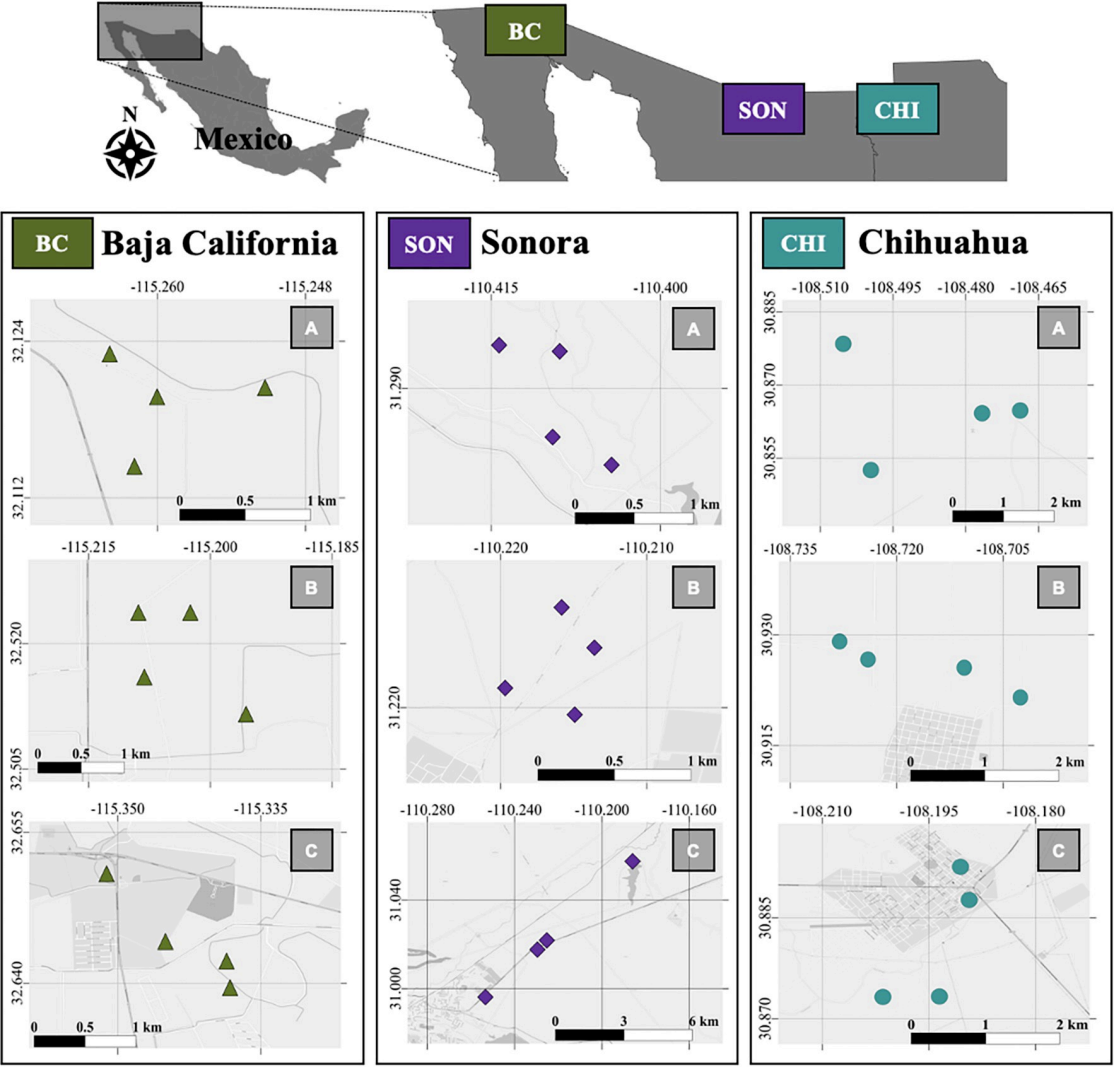
Map of Northwestern Mexico and the study sites. The buffers that correspond to Baja California are shown in green, Sonora in purple, and Chihuahua in cyan. Boxes represent sites characterized by predominant native (A) vegetation, (B) cropland or livestock, and (C) urban/peri-urban areas. Reprinted from the OpenStreetMap vector basemap hosted by Environmental Systems Research Institute (Esri) and provided under a CC BY 4.0 license (Map data © OpenStreetMap contributors, Map layer by Esri 2019).

This study was conducted in three regions of Northwestern Mexico: Mexicali, Baja California (BC); Janos-Monte Verde, Chihuahua (CHI); and San Pedro River Basin-Cananea, Sonora (SON) (Fig 1). Each region represents a SES since they are composed of different levels of landscape heterogeneity and different types and levels of human activities, allowing us to compare anthropization degrees between sites [33–35]. One of the main features of these SESs is represented by the Mexico-US borderline, which is characterized by high levels of urban transformation, agricultural and farming intensification, and industrial activities that represent important pressures on the environment [35–37]. BC region is a semi-desert zone dominated by urban matrices with the presence of xerophytic and sclerophyllous scrub, followed by areas for agricultural exploitation [38]. The predominant vegetation type in CHI is mainly composed of native grasslands, followed by mesquite, riparian vegetation, and oak forest, as well as land-use for agricultural activities [34]. Vegetation in SON is characterized by native grasslands, followed by marshes, riparian corridors, and oak savannas [39]. In addition, previous studies have identified the presence of zoonotic pathogens and their related reservoirs, as in the case of rodent species and Hantavirus [40, 41], Arenavirus [42], *Bartonella* spp. [43], *Rickettsia* spp. [44], and *Coccidioides* spp. [45]. For each region, we established 12 sites separated by at least 500 m from each other, for a total of 36 sites (Fig 1; see S1 File for geographic coordinates).

### Monitoring of rodent reservoirs

To evaluate reservoir communities, we captured peridomestic and wild rodents at the 36 study sites. For rodent capture, we placed a grid of 7x7 Sherman traps in the center of each study site, remaining active for three consecutive nights. All rodent captures and handling procedures followed the regulations of the *Subcomité Interno para el Cuidado y Uso de los Animales de Experimentación* (Institutional Animal Care and Use Committee) No. SICUAE.DC-2021/2-7 of the Universidad Nacional Autónoma de México (UNAM). We used isofluorane for rodent management according to the recommendations of the American Society for Mammalogy guidelines for the use of wild mammals in research [46]. No animals were euthanized in this study. In addition, we followed standard safety guidelines recommended by the Center for Disease Control and Prevention [47]. The captured rodents were taxonomically identified with field guides [48, 49], tagged with earrings for individual identification, and released at the exact site of capture. Subsequently, we identified the status of each species as a reservoir or no-reservoir of zoonotic pathogens, following the categorization published by Han et al. [16, 50]. In each region, the monitoring was conducted in two seasons, corresponding to winter 2020 and summer 2021 for BC and CHI, and autumn 2019 and spring 2022 for SON. Due to logistical issues related to insecurity in the study area as well as the onset of the COVID-19 pandemic, it was not possible to homogenize the sampling seasons for the three regions, but only for BC and CHI.

### Anthropization degree

To assess the anthropization degree, we developed an Anthropization Index by conducting geographic, ecologic, socioeconomic, and demographic analyses using measuring variables to determine the changes produced by human activities in different aspects, scales, and dimensions within a SES (Table 1). We evaluated a total of 15 variables, classified into five major groups: Vegetation type, Urbanization, Habitat fragmentation, Water quality, Potential contaminant sources, and Others (Table 1; see S1 File for more detail). The variables related to anthropization used in this study are or can be associated with the dynamics of infectious diseases by altering the prevalence of a pathogen and/or its reservoirs by 1) changing local biodiversity, 2) increasing coexistence between humans and reservoirs, and 3) promoting social processes related to disease burden [51–54].

**Table 1:** Anthropization variables. We evaluated 15 variables classified into five groups. Most of the variables were derived from repositories and databases.

Classification	Variable	Description	Reference
Vegetation type	Non-native vegetation^a^	Determined by the total area of any non-native vegetation in the impact area.	13, 54, 56, 57
Urbanization	Light pollution^a^	Urbanization indicators are given by anthropogenic night-time light. The urban limit is given by a Radiance equal to 30x10^−9^Wcm^−2^sr^−1^. We added the pixels within the impact area and took the average of the total Radiance.	53, 58, 59
	Distance to the nearest urban core	Inverse multiplicative of linear distance between the impact area center and the two nearest pixels with Radiance equal to or greater than 30x10^−9^Wcm^−2^sr^−1^.	54, 58–60
	Distance to the second-nearest urban core		
	Urban buildings^a^	Percentage of impact area covered by residential, commercial, or industrial developments.	5, 55
Water quality	Microbiological water quality^a^^,^^b^	Surface water samples were taken from water bodies in the impact zones. The amounts (colony-forming unit count) of Total Coliforms and *E*. *coli* were evaluated using 3M^TM^ Petrifilm^TM^ plates as culture medium, following the specifications recommended by the manufacturer. Microbiological quality was rated as the average of colonies in water samples taken from bodies in each impact zone. We took three samples of 1 ml each from at least three water bodies at each site. The samples were taken from the center, the edge, and one-third from the center at a depth of 30 cm in small water bodies and 1 m in the case of rivers, ponds, and dams.	31, 61–64
	Chemical water quality^a^^,^^b^	We evaluated the concentration of three different compounds (Nitrate, Ammonia and Orthophosphates concentration) that can indicate excess nutrients in water samples (contamination). We used optical spectrophotometry and averaged the results of all samples within a site for rating. We used the same samples of microbiological water quality, but with samples of 100 ml.	31, 62–65
Potential contaminant sources	Landfill proximity	Inverse multiplicative of linear proximity of impact zones to the nearest potential contaminant source.	5, 31
	Gas station proximity		
	Mining activities proximity		
	Hospital proximity		
	Graveyard proximity		
Other	Human population density^a^	Number of people divided among the total area covered by a buffer of r = 500 m.	12, 60, 66
	Roads and highways^a^	Roads and highways network surface area between buffer surface area.	5, 67–69
	Livestock activity^a^^,^^b^	We obtained the average of three counts of the number of cows, horses, goats, sheep, pigs, and poultry present at the study sites at the time the rodent traps were checked and over a period of 10 minutes.	51, 70–72

^a^ Variable measured in buffers of 500m as the impact zone.

^b^ Variables analyzed *in situ*.

To evaluate some of the variables, we established buffers of 500 m radius as the impact area (see Table 1 for specific variables). The centers of the buffers correspond to the capture grids placed at the 36 sites. The analysis was performed with the free geographic information system QGIS version 3.10.10-A Coruña (2018, Free Software Foundation, Inc.), based on LandSat8 satellite images, geospatial data repositories, official databases, and field data collection (Table 1) [54–72]. To obtain the Anthropization Index, we performed a Principal Component Analysis (PCA) to combine the variables into a new variable named ‘anthropization degree’, as well as to identify which types of human activities impact more in each locality and what the intensity of their effect is [54, 55]. To perform PCA, we used the ‘princomp’ function from the ‘stats’ package in R Studio (2020, RStudio Team, Inc.). We set the anthropization degree as the scores belonging to the first Principal Component (PC) in our analysis since PCA scores are the representation of a linear combination of values recorded for each metric (anthropogenic variables) [54]. To establish an index, each score was rescaled to a range between 0 and 1 using the ‘rescale’ function from the ‘scales’ package in R Studio, where 0 corresponds to the value of the least anthropized site and 1 to the value of the most anthropized site.

### Effect of anthropization on the rodent community

The response of rodents to anthropization was evaluated by assessing the association between relative abundance (RA, proportion of individuals over the total abundance) and richness (number of species) of reservoir and non-reservoir rodents and the degree of anthropization, as estimated by our index. Because sampling was conducted in different seasons in one of the regions, we grouped BC and CHI (BC+CHI) and analyzed SON independently. Generalized Linear Mixed Models (GLMM) were performed, setting RA and richness as dependent variables in separate models. In both sets of models, we included ‘anthropization degree’ as the independent variable as well as ‘sampling season’. The region was included as a variable only for the BC+CHI models. All models also included ‘site’ as a random effect to account for the local variation at each trap station [73]. Because anthropization is not likely to exhibit a homogeneous effect among species and/or between individuals of the same species, we conducted further analyses using the two most abundant rodent species captured in all sampling seasons (*M*. *musculus* and *Peromyscus maniculatus*), which are also species recognized as important reservoirs of zoonotic pathogens [50]. We modeled the RA as a proportion using a binomial error distribution and a Poisson distribution to model the effect of anthropization on the richness of reservoirs and non-reservoirs and the abundance of *M*. *musculus* and *P*. *maniculatus*. We used the ‘glmer’ function from the ‘lme4’ package to fit GLMMs, and the “check_overdispersion” function from the ‘performance’ package to assess for overdispersion in Poisson models. We used the ‘ggeffects’ package to visualize model predictions of RA and richness models in the case of BC+CHI. All analyses were performed in R Studio (2020, RStudio Team, Inc.).

## Results

### Rodent reservoir community

A total of 683 individuals of 27 species were captured, representing 14 genera and four families in the three regions from September 2019 to April 2022 (Table 2; S1 File). In total, nine rodent species were identified as reservoirs of zoonotic pathogens: *Baiomys taylori*, *M*. *musculus*, *Neotoma albigula*, *P*. *eremicus*, *P*. *leucopus*, *P*. *maniculatus*, *R*. *rattus*, *Reithrodontomys megalotis*, and *Sigmodon hispidus* (Table 2). In BC, five reservoir species were identified from the 17 species captured in both seasons, while in CHI and SON, six reservoirs were identified in each region from the 17 and 14 species captured, respectively. In the case of reservoir abundance, 57 reservoirs from 157 individuals (36%) were identified in BC in both seasons, 99 reservoirs from 234 individuals (42%) in CHI, and 189 reservoirs from 292 individuals (64%) in SON (Table 3).

**Table 2:** List of rodent species captured in the survey. Number of individuals of each species for each region and both sampling seasons.

Region	Species	Abundance	
		First season^a^	Second season^a^
BC	*Chaetodipus baileyi*	0	1
	*Chaetodipus penicillatus*	14	51
	*Chaetodipus spinatus*	0	7
	*Dipodomys merriami*	4	3
	*Mus musculus* ^b^	4	12
	*Neotoma albigula* ^b^	0	2
	*Peromyscus fraterulus*	14	3
	*Peromyscus maniculatus* ^b^	30	4
	*Rattus rattus* ^b^	1	1
	*Reithrodontomys megalotis* ^b^	3	0
	*Xerospermophilus tereticaudus*	2	1
	Total	72	85
CHI	*Chaetodipus eremicus*	0	2
	*Chaetodipus hispidus*	2	0
	*Chaetodipus intermedius*	7	11
	*Dipodomys merriami*	31	8
	*Dipodomys ordii*	7	1
	*Dipodomys spectabilis*	2	3
	*Mus musculus* ^b^	5	9
	*Neotoma albigula* ^b^	1	6
	*Onychomys arenicola*	29	5
	*Onychomys leucogaster*	9	1
	*Perognathus flavus*	14	0
	*Peromyscus eremicus* ^b^	8	0
	*Peromyscus leucopus* ^b^	13	1
	*Peromyscus maniculatus* ^b^	45	4
	*Reithrodontomys fulvescens*	1	0
	*Sigmodon hispidus* ^b^	6	1
	*Xerospermophilus spilosoma*	0	2
	Total	180	54
SON	*Baiomys taylori* ^b^	1	0
	*Chaetodipus hispidus*	27	9
	*Chaetodipus intermedius*	8	0
	*Chaetodipus penicillatus*	4	3
	*Mus musculus* ^b^	70	87
	*Onychomys leucogaster*	0	1
	*Onychomys torridus*	9	1
	*Perognathus flavus*	1	37
	*Peromyscus leucopus* ^b^	7	0
	*Peromyscus maniculatus* ^b^	1	12
	*Rattus rattus* ^b^	4	1
	*Reithrodontomys montanus*	1	0
	*Sigmodon hispidus* ^b^	4	2
	*Xerospermophilus spilosoma*	0	2
	Total	137	155

^a^ For BC and CHI, the first and second seasons correspond to winter 2020 and summer 2021, respectively. For SON, the first and second seasons correspond to autumn 2019 and spring 2022, respectively.

^b^ Reservoir species.

**Table 3:** Richness and abundance of rodents. Reservoir species and individuals’ percentages of the total number of species and individuals captured are shown in parenthesis.

Region	Sampling season	Species richness	Number of individuals	Reservoir richness	Reservoir abundance	*M*. *musculus* abundance	*P*. *maniculatus* abundance
BC	Winter 2020	8	72	4 (50%)	38 (53%)	4 (10.5%)	30 (79%)
	Summer 2021	10	85	4 (40%)	19 (22%)	12 (63%)	4 (21%)
CHI	Winter 2020	15	180	6 (40%)	78 (43%)	5 (6.4%)	45 (57.7%)
	Summer 2021	13	54	5 (38%)	21 (40%)	9 (43%)	4 (19%)
SON	Autumn 2019	11	136	6 (54%)	87 (64%)	70 (80%)	1 (1.1%)
	Spring 2022	11	156	4 (36%)	102 (65%)	87 (85%)	12 (11.8%)

### Anthropization degree

**Fig 2:**
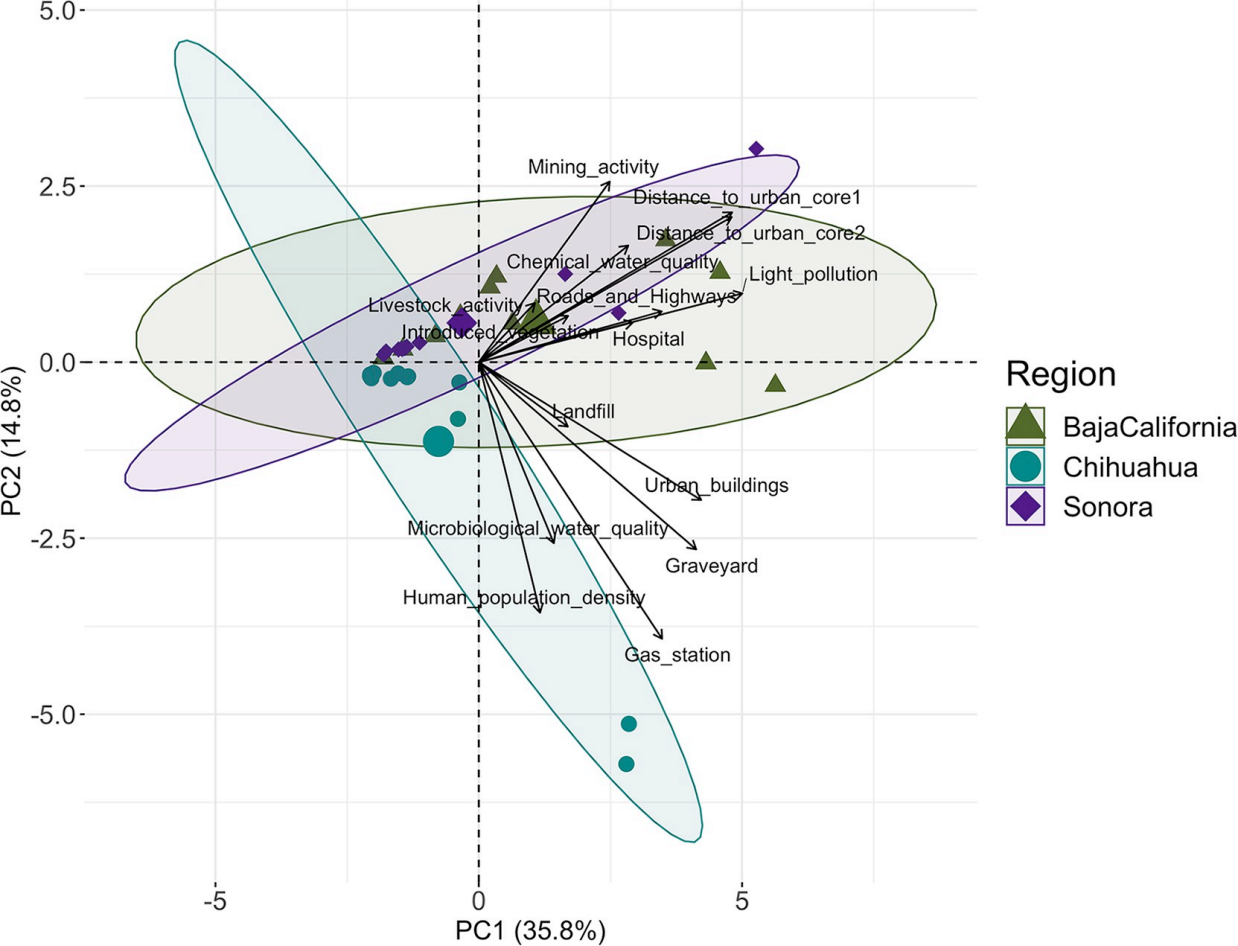
PCA-Biplot of variables and sites. The small symbols represent the sites in each region and are contained in an ellipse, representing the 95% Confidence Intervals (CI) for each region. The larger, centered symbols in each ellipse represent the average of the scores for each region.

The sites analyzed showed different anthropization scores and followed anthropization gradients according to their region (Fig 2). For example, anthropization scores from sites in the CHI region were lower compared with sites in BC (S1 File). The first two PCs of the PCA explained more than 50% of the data variance (35.8% and 14.8%, respectively) (Fig 2), and the variables measured had different contributions to the development of the Anthropization Index (S1 Fig). ‘Light pollution’, ‘Distance to the nearest human core’, ‘Distance to the second-nearest human core’, and ‘Urban buildings’ variables had the strongest influence in the first PC (from which we obtained the scores), while ‘Livestock activity’, and ‘Human population density’ variables had less influence on the Anthropization Index (S1 Fig).

### Association between anthropization and rodents

**Fig 3:**
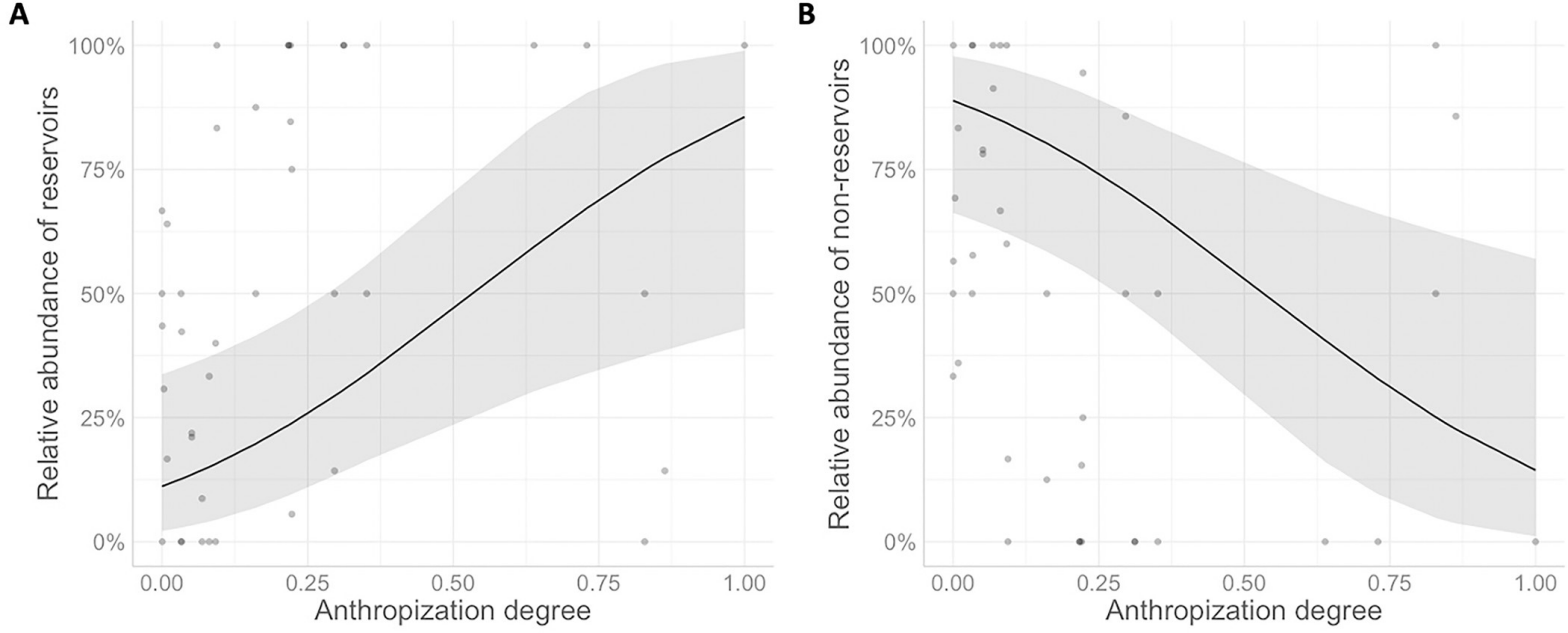
GLMM’s prediction plots of the effect of anthropization on the RA of rodents. (A) Predicted probabilities for reservoir species and (B) predicted probabilities for non-reservoir species in BC+CHI. Grey shadows represent CI 95%.

We found a positive and significant association between anthropization and the RA of zoonotic reservoirs for both BC+CHI and SON groups (p = 0.007 and p<0.001, respectively) (Table 4, Fig 3), where the RA of reservoirs increased at higher degrees of anthropization. We also detected a seasonal effect in BC+CHI regions, where the RA was higher in winter compared to summer (p<0.001), but there was no significant difference in RA between regions in this group (p = 0.311). No seasonal differences were detected in the SON region (p = 0.795) (Table 4). On the other hand, the RA of non-reservoir species decreased significantly at higher anthropization scores for both models (B+C, p = 0.007; SON, p<0.001) (Table 4, Fig 3). For BC+CHI, the RA of non-reservoirs was lower in winter compared to summer (p<0.001), while there was no association with regionality for this group (p = 0.311) or with seasonality in the SON region (p = 0.795) (Table 4).

**Table 4:** Results of the GLMMs to test for the association between RA and richness of reservoir and non-reservoir species. The model output includes the estimates for the fixed and random effects. Numbers in bold indicate significant values.

Model	Regiion	Anthropization variable			Region variable			Season year variable			Site (random effect)	
		Estimate	SE	p-value	Estimate	SE	p-value	Estimate	SE	p-value	Var	SD
RA of reservoirs	BC+CHI	2.280	0.851	**0.007**	Chihuahua = 0.438	0.433	0.311	Winter2020 = 0.796	0.180	**<0.001**	0.590	0.768
	SON	5.021	0.721	**<0.001**	N/A	N/A	N/A	Spring2022 = -0.066	0.256	0.795	0.242	0.492
RA of non-reservoirs	BC+CHI	-2.280	0.851	**0.007**	Chihuahua = -0.438	0.433	0.311	Winter2020 = -0.796	0.180	**<0.001**	0.590	0.768
	SON	-5.021	0.721	**<0.001**	N/A	N/A	N/A	Spring2022 = 0.066	0.256	0.795	0.242	0.492
*M*. *musculus* abundance	BC+CHI	4.866	2.127	**0.022**	Chihuahua = 0.323	1.258	0.797	Winter2020 = -0.847	0.398	**0.033**	3.546	1.883
	SON	8.638	2.074	**<0.001**	N/A	N/A	N/A	Spring2022 = 0.217	0.160	0.175	1.760	1.327
*P*. *maniculatus* abundance	BC+CHI	-6.737	1.671	**<0.001**	Chihuahua = -0.529	0.379	0.163	Winter2020 = 2.238	0.371	**<0.001**	0.249	0.499
	SON	2.784	2.116	0.188	N/A	N/A	N/A	Spring2022 = 2.485	1.041	**0.016**	2.121	1.456
Reservoir richness	BC+CHI	-1.088	0.609	0.074	Chihuahua = 0.179	0.308	0.560	Winter2020 = 0.575	0.294	0.051	0	0
	SON	2.329	0.753	**0.001**	N/A	N/A	N/A	Spring2022 = -0.693	0.433	0.109	0.129	0.360
Non-reservoir richness	BC+CHI	-2.746	0.818	**<0.001**	Chihuahua = -0.274	0.385	0.477	Winter2020 = 0.111	0.236	0.637	0.290	0.539
	SON	-0.836	0.660	0.205	N/A	N/A	N/A	Spring2022 = -0.046	0.305	0.878	0	0

SE, standard error; Var, variance; SD, standard deviation; N/A, not applicable.

**Fig 4:**
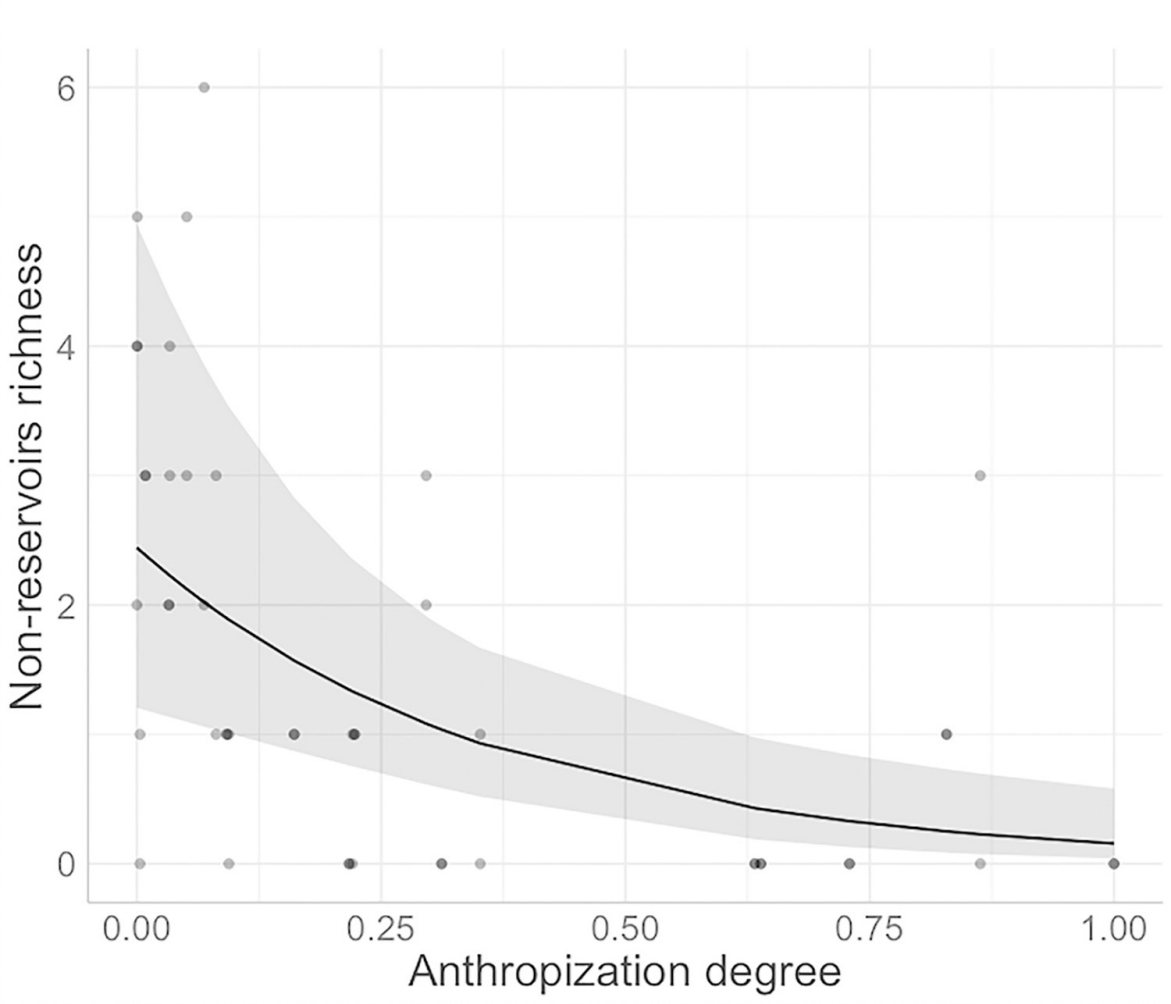
GLMM’s prediction plot of rodent richness. Predicted counts of the association between anthropization and non-reservoir richness in BC+CHI. Grey shadows represent CI 95%.

In BC+CHI, we found a negative but marginally significant association between anthropization and reservoir richness (p = 0.074) (Table 4). For the same region group, the winter season had higher reservoir richness compared to the summer season (also with a marginally significant association, p = 0.0508), while there was no difference between regions of the same group (p = 0.560) (Table 4). In the SON region, there was a significant positive association between reservoir richness and anthropization (p = 0.001), while there was no association with the sampling season (p = 0.109) (Table 4). We found that non-reservoir species richness was negative and significantly associated with anthropization in BC+CHI (p<0.001) (Fig 4), but not between regions and seasons in the same group (p = 0.477 and 0.637, respectively) (Table 4). In SON, there was no association between non-reservoir richness and anthropization or seasons (p = 0.205 and 0.878, respectively) (Table 4).

When we individually analyzed *M*. *musculus*, the association between abundance and anthropization was positive and significant for both BC+CHI and SON groups (p = 0.022 and p<0.001, respectively) (Table 4). *M*. *musculus* abundance was lower in winter compared to summer for BC+CHI (p = 0.033), while there was no difference between regions in this group (p = 0.797) or between seasons in SON (p = 0.175) (Table 4). When we analyzed *P*. *maniculatus* separately, we found a negative and significant association between abundance and anthropization in BC+CHI (p<0.001) but not between regions of the same group (p = 0.163) (Table 4). In addition, we found that there was a significant difference between seasons, where abundance was higher in winter compared to summer for the BC+CHI group (p<0.001) (Table 4). For SON, there was no association between *P*. *maniculatus* abundance and anthropization (p = 0.188), but there was a significant difference between seasons with a higher abundance in spring compared to autumn (p = 0.016) (Table 4). No overdispersion was detected for any of the analyses (S1 Table). The variance of the random effect indicated variation in RA and richness of reservoir and non-reservoir species between trap sites, but this depended on the variable assessed and the region (Table 4).

## Discussion

Our results show that the RA and richness of rodent reservoirs of zoonotic pathogens increase with anthropization, while the RA and richness of non-reservoir rodent species tend to decrease (Table 4). An increase in the RA of rodent reservoirs can constitute a risk for zoonoses outbreaks by enhancing the contact rate among reservoirs and potential hosts, including humans, in density-dependent disease systems such as hantavirus [74]. Although the association between anthropization and reservoir and non-reservoir richness varied between regions, the increase of reservoir richness in SON and the decrease of non-reservoir species in BC+CHI may result in the loss of the local dilution effect and favor the transmission of zoonotic pathogens between competent hosts [75]. Similarly, the decreases in richness and RA of non-reservoir rodents under anthropization pressures have implications for biodiversity conservation, mainly because anthropization promotes environmental filtering that induces such species (usually non-generalist) to become locally extinct [76].

However, we found that the impact of anthropization degree among reservoir species was not homogeneous. For *M*. *musculus*, an invasive commensal and reservoir species [77], anthropization clearly increases its abundance in both groups of regions, whereas the abundance of *P*. *maniculatus*, a reservoir species with native distribution [78], decreases as anthropization increases in BC+CHI. This is potentially due to the different ecological traits of each species; although both share traits that make them excellent reservoirs (e.g., high number of litters per year or sexual maturity at a young age) [16, 18], they respond differently to anthropogenic disturbance. Our findings are consistent with the results of Ecke et al. [79], who demonstrated that the fluctuations of rodent reservoir populations depend on species synanthropy and the degree of human exploitation. For example, *M*. *musculus* is recognized as a species that directly benefits from human activities in urban and peri-urban areas by obtaining food and shelter [80, 81], while *P*. *maniculatus* is more common in croplands [82]. Likewise, the transmission of their related zoonotic pathogens to people occurs through different scenarios: *M*. *musculus* tends to transmit pathogens due to high coexistence with humans (e.g., inside a house by contaminating food with urine and feces) [15, 24], while pathogens transmitted by *P*. *maniculatus* are often transmitted to people with outdoor-related activities, such as working in fields or forested areas, or recreational activities in natural areas [83, 84].

While there was not any difference in RA between regions in BC+CHI, our results showed that there are seasonal differences influencing the RA of rodents. For the winter of BC+CHI, there was a slight increase in the RA of reservoir species and a decrease in the RA of non-reservoir species. This was probably because reservoir species, usually generalist species, can obtain food from human activities at the more anthropized sites (where they were more abundant in this study), while non-reservoir species tend to be non-generalist species that are compromised by a lack of resources during the winter [85, 86].

In the analysis for *M*. *musculus* and *P*. *maniculatus*, we also found differences between sampling seasons. In BC+CHI, the abundance of *M*. *musculus* was slightly lower in the winter compared to the summer. This effect has been reported in *M*. *musculus* because reproductive strategies can be modified when temperature declines in winter [87, 88]. Although there was no difference between seasons in SON, the high abundance of *M*. *musculus* was recorded in both seasons at the most anthropized site in this region (S1 File). This site (BCurb1) is located next to the municipal landfill of Cananea, BC, which has grown approximately 50 m in diameter since the COVID-19 pandemic began, a phenomenon shared in other regions of the world [89], and probably due to the increase in plastic waste such as gloves and masks used as Personal Protective Equipment [90]. Landfills have been proven to unintentionally provide food for generalist and synanthropic species that can be excellent reservoirs of zoonotic pathogens [91], which may explain the case of *M*. *musculus* in SON. Consistently, two studies have found an increase in the movement and abundance of commensal rodents following the onset of the COVID-19 lockdown in New York [92] and Sydney [93]. These changes may potentially promote the spread of pathogens transmitted by *M*. *musculus*, *R*. *rattus*, and other invasive rodents.

For *P*. *maniculatus*, the winter in BC+CHI and the spring in SON had higher abundance compared to summer and autumn, respectively (Table 4). These changes can be explained by the fact that the increase (or decrease) in the abundance of granivorous rodents, such as *P*. *maniculatus*, may be linked to the decrease (or increase) of other species due to the availability of resources [94]. In our survey, the abundance of *P*. *maniculatus* in the three regions increases in seasons when the abundance of *Chaetodipus* spp. decreases (S1 File).

Changes in rodent communities in arid environments, as in the case of Northwestern Mexico, could also be linked to naturally occurring population fluctuations due to seasonal changes in rainfall and resource availability [95, 96]. However, our model structure allowed us to control for the effects of local variation in trapping stations [73], as well as the potential effects of interannual seasonality. In addition, because SON did not share the same sampling seasons with the other regions, we were restricted from evaluating the effect of regionality with a more statistically robust approach.

The Anthropization index developed from the PCA proved to be a reliable approach to characterizing the landscape since the least anthropized sites corresponded to conserved and rural localities in each region, while the most anthropized sites corresponded to urban-periurban localities (Figs 1 and 2). In fact, the variables that most influenced the Anthropization Index were directly related to urbanization, such as ‘Light pollution’ and ‘Distance to the nearest human core’ (S1 Fig), which have also been associated with a heightened risk of pathogen transmission. For example, the ’Light pollution’ variable has been previously related to changes in mosquito biting patterns with potential for arbovirus transmission [53] and can also modify mobility patterns and other life history traits in several reservoir and non-reservoir species [59], altering their community structure. The strong influence of urban variables is related to the high rate of land-use change due to agriculture, livestock, and urbanization in the region [33, 97]. It has been highlighted that urbanization may favor the development of future pandemics of zoonotic origin in the Anthropocene [4, 8], due to rapid human population increase, high human population density, high rate of human movement, the dominance of commensal species that serve as reservoirs of zoonotic pathogens (such as *Rattus spp*. and *M*. *musculus*), and socioeconomic disparity [98–100]. For example, Chaisiri et al. [27] found higher helminth richness in *Rattus spp*. in sites with greater human disturbance, while Prist et al. [101] found that the decrease in anthropization from reforestation decreases the abundance of rodent reservoirs of hantavirus [91]. Therefore, monitoring the RA of rodent reservoirs in urban and peri-urban areas is of utmost importance to identify potential zoonotic outbreak risk hotspots [5, 102].

The development of new quantitative tools, such as indexes, can allow us to study complex systems by simplifying the analysis of multiple variables and large-scale data. The Anthropization Index that we developed could be useful to assess the effect of human activities not only in eco-epidemiological surveys but also in conservation programs.

## Conclusion

In the so-called Anthropocene [103], the continuous influence of human activities on almost all Earth processes has caused, among many other things, an increased risk of zoonotic outbreaks. This complex phenomenon can only be approached from a holistic perspective that allows the inclusion of different perspectives and analyses, as in the case of the One Health approach. For that reason, in this study, we used the concept of SES to define our scale, which allowed us to go deeper by considering that all natural and human-induced processes are connected. Moreover, analyzing the association between anthropization and rodent reservoirs of zoonotic diseases allowed us to examine the interconnected aspects of animal health, environmental health, and human health. Our results are consistent with theoretical and empirical studies that have found that anthropization increases the abundance of reservoir rodents and decreases the abundance of species that have not been reported as reservoirs. However, the response of reservoir species to anthropization was heterogeneous, and it would be important to consider the species identity to analyze rodent populations specifically. Given the prevalence of anthropogenic impact at the local, regional, and global scales and the proliferation of reservoir species associated with anthropogenic pressure, our results are of broad significance and warrant further research in other locations of the world.

## Supporting information

S1 File Database of a) site coordinates, b) Anthropization Index variables, c) anthropization degree of sites, and d) rodent monitoring.
(XLSX)

S1 Table Overdispersion evaluation for GLMM with Poisson distribution. (CSV)

S1 Fig Variables contribution plot. Contribution of the six variables that contributed the most in PC1. The red dashed line indicates the expected average contribution.
(TIFF)
