# Supplementary figures and images for: Association between anthropization and rodent reservoirs of zoonotic pathogens in Northwestern Mexico
Source: PLoS One. 2024 Feb 22;19(2):e0298976. doi: 10.1371/journal.pone.0298976 (PMC10883555; doi:10.1371/journal.pone.0298976)

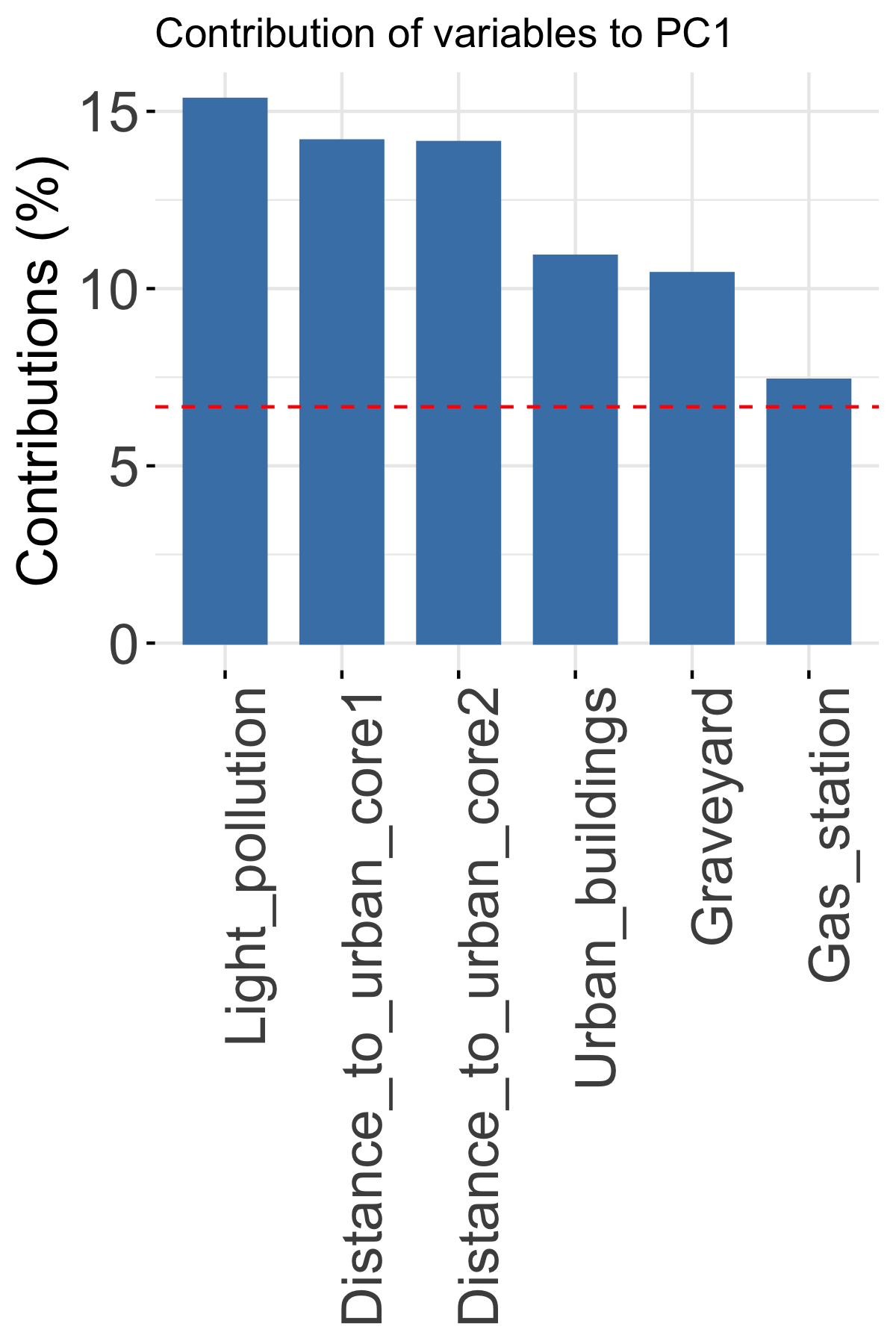

Supplement: S1 Fig — Contribution of the six variables that contributed the most in PC1. The red dashed line indicates the expected average contribution. (TIFF) [file pone.0298976.s003.tiff]
